# Organization of rehabilitation services for youth with physical disabilities and mental health problems: A scoping review

**DOI:** 10.3389/fresc.2023.1085827

**Published:** 2023-02-20

**Authors:** Stephanie Tremblay, Shalini Lal, Lucille Xiang, Mark A. Ferro, Dana Anaby

**Affiliations:** ^1^ School of Physical and Occupational Therapy, McGill University, Montreal, QC, Canada; ^2^ PEPP Montreal and ACCESS Open Minds, Douglas Mental Health University Institute, Montreal, QC, Canada; ^3^ Center for Interdisciplinary Research in Rehabilitation of Greater Montreal (CRIR), Montreal, QC, Canada; ^4^ School of Rehabilitation, University of Montreal, Montreal, QC, Canada; ^5^ Health Innovation and Evaluation Hub, University of Montreal Hospital Research Center, Montreal, QC, Canada; ^6^ School of Public Health, Brown University, Providence, RI, United States; ^7^ School of Public Health Sciences, University of Waterloo, Waterloo, ON, Canada

**Keywords:** service organization, rehabilitation, co-occurring diagnoses, mental health services, service delivery

## Abstract

**Introduction:**

Youth with childhood-onset physical disabilities receiving rehabilitation services often present with many complex needs. Emerging evidence confirms co-occurrence of mental health problems in this population is common, and mental health is often overlooked during rehabilitation for chronic physical conditions. For example, symptoms of depression and anxiety are frequently present in adolescents with physical disability such as spina bifida or Duchenne muscular dystrophy, and access to mental health services is often limited. Addressing mental health concerns for this age group is particularly critical as it encompasses a challenging transition to adulthood.

**Objectives:**

Building upon findings from a recent scoping review on the co-occurrence of physical disabilities and mental health problems, this paper synthesizes scientific literature related to the organization and delivery of services for youth with co-occurring childhood-onset physical disabilities (e.g., cerebral palsy, spina bifida) and mental health problems (e.g., depression, anxiety).

**Methods:**

A scoping review protocol stemming from Arksey & O'Malley's framework and updated guidelines from the Joanna Briggs Institute was developed. Four databases (Medline, PsycINFO, CINAHL, Embase) were searched. The search was limited to French or English peer-reviewed articles published between 2000 and 2021. Articles included were primary papers addressing: 1) youth aged 15 to 24 with a childhood-onset physical disability, 2) mental health problems, and 3) healthcare service organization or delivery. They were screened by two reviewers and discussed with a third to establish consensus on the inclusion criteria and resolve disagreements.

**Results:**

Sixteen articles were retained from the 1,010 screened. Many (9/16) were from the United States. Two models were found: the Biopsychosocial, Collaborative, Agency-Based Service Integration Approach (including psychiatry in a pediatric rehabilitation hospital) and the Client Network Consultation (an interagency collaboration in mental health care for children with complex healthcare needs). Twelve key principles for service organization and delivery were identified and categorized into: collaboration and coordination, training and support, and delivery of care.

**Conclusion:**

Identified principles can guide improved service delivery for this population. Highlighted research gaps include the need for developing models of collaborative healthcare delivery and subsequently evaluating their effectiveness.

## 1. Introduction

Approximately 20% of Canadians will develop a mental illness before the age of 25, and less than one fifth will receive appropriate and timely treatment (1, 2). Youth with childhood-onset physical disabilities (such as cerebral palsy) who receive rehabilitation services often present with many complex needs, and there is emerging evidence that co-occurrence of mental health problems in this population is common (3–5). A large Canadian study (*n* = 5947) examining prevalence found that physical (respiratory, musculoskeletal, cardiovascular, neurological, endocrine/digestive) and mental (depression, suicidal behaviour, bipolar disorder, generalized anxiety disorder) comorbidity is common among young adults (6). Additionally, mental health is often overlooked when clients are treated for chronic physical conditions (3). A recent scoping review carried out by our team (4) also revealed that symptoms of depression and mood-related difficulties, followed by anxiety and social and behavioral difficulties are frequently present in adolescents and young adults with physical disability such as spina bifida and Duchenne muscular dystrophy. Despite the high occurrence of mental health disorders, symptoms or behavioral problems, few studies included in the review addressed access to, and organization of, mental health services. Addressing mental health concerns for this targeted age group is particularly critical as this life stage encompasses the transition to adulthood which brings about its own set of challenges.

Integrated care is generally considered to be ideal, yet effective strategies and principles for organizing and delivering services for youth with co-occurring physical and mental disorders have not been established (7). Indeed, little is known about how health and rehabilitation services address the mental health needs of youth with childhood-onset physical disabilities. Thus, our team first carried out a scoping review to explore the rates of co-occurrence of mental health problems among adolescents and young adults with a physical disability, and also to describe if they had access to mental health care (4). Only 10 out of the 33 retained articles addressed access to services, which led to four related themes: use, access, and experiences of mental health services; stigma; mental health of family caregivers; and the value of comprehensive mental health services. Few participants who had mental health concerns accessed services, and many unmet needs were reported. Despite a push for more, or better access to, mental health services for those with physical disabilities, no article from that scoping review detailed how such services could be organized to fit with existing systems of care (4). Therefore, it was established that the cooccurrences were prevalent, but it remained unclear how services were organized and delivered. To address this, a subsequent literature review including broader publication dates (between 2000 and 2021 vs. 2007–2019 in the first scoping review) and a more specific search strategy (focused on the organization of services themselves, with additional keywords such as service delivery and service integration) was proposed. The additional inclusion of children with special healthcare needs ensured that articles addressing a variety of physical/chronic disabilities within rehabilitation were taken into consideration.

Healthcare services in many high-income countries are typically rooted in a biomedical model and are not presently prepared to meet the psychosocial needs of children and youth with physical disabilities (8, 9). In Canada, there are steps being taken towards integrating mental health into primary care (though as of now it remains at the level of some organizations and provinces), while other countries such as England have put in place specialized programs (such as the Increasing Access to Psychological Therapies program) to address this challenge (10). In the United States of America (United States), mental health services are not yet universally accessible either (10). More specifically, mental health care is not well integrated in rehabilitation services across countries (11). In response to the gap, the World Health Organization (WHO) has recently come up with the special initiative for mental health (2019–2023) document (12) which aims to achieve universal mental health coverage for all, focusing in particular on low-income countries and remote regions where access to care is especially complex. Its second strategic action, which promotes the scaling up of interventions and services across community-based, general health and specialist settings, includes ensuring that affordable, quality mental health care is integrated in relevant programs such as in rehabilitation care for people with disabilities, and stipulates that priority efforts are needed to integrate mental health care across all levels (12).

A better understanding of the organizational contexts of various healthcare systems and efficient models of mental health service delivery would be pertinent to ensure that the complex needs of this population are met more comprehensively. The current context of healthcare service reorganization, coupled with pressures deriving from budget cuts and increased need and demand for services, provides a timely opportunity to study actual health and rehabilitation services, to outline needs and to identify gaps to inform future service delivery methods and ensure that best practices are translated into clinical settings (13, 14).

The objective of this review is to identify what is known about existing health and rehabilitation services and models of service delivery for youth with co-occurrence of childhood-onset physical disabilities and mental health challenges.

## 2. Materials and methods

A scoping review methodology was selected to answer the broad research question by mapping the available evidence since existing models and services available for this population have not yet been comprehensively reviewed. Stemming from Arksey & O'Malley's framework (15) and the recommendations provided by Levac and colleagues (16), Colquhoun and colleagues (17) and the JBI Manual for Evidence Synthesis: Scoping Review chapter from the Joanna Briggs Institute (18), a detailed protocol was created and registered in Open Science Framework online in December 2021 (19). This scoping review includes 6 stages: identifying the research question, identifying relevant studies, selecting studies, charting data, collating results, and consultation. A preliminary meeting was held with 3 stakeholder clinicians to contribute ideas pertaining to additional literature and review the search strategies. One clinician from a physical rehabilitation team agreed to serve as a member of a consultation committee to help interpret and validate the results and assist in dissemination.

Scientific evidence obtained from peer-reviewed journal articles *via* Ovid Medline, CINAHL, PsycINFO and Ovid Embase was targeted. These four databases were chosen as complementary to encompass the inclusion criteria and retrieve the highest possible number of publications in the disciplines of biomedicine and psychological sciences (20). A university-based librarian with expertise in rehabilitation was consulted when selecting the databases and finalizing the search strategy.

### 2.1. Identifying the research question

Using the PCC framework (Population/Participants, Concept, Context) and building on the previous scoping review findings (4), this review aimed to answer the following research question: What is known about existing health and rehabilitation services and models of service delivery for youth with co-occurrence of childhood-onset physical disabilities and mental health problems? Additional sub-questions include: 1) How are these services organized and delivered? 2) What are the key principles of existing models of service delivery? 3) What is known about the effectiveness of identified models or principles?.

### 2.2. Identifying relevant studies

The search terms included four topics and their derivatives: physical disability, mental health disorder, youth, and healthcare service. The initial search strategy was developed for Medline, building on previous scoping reviews (4, 21) and as seen in Table 1. It was then adapted to the other databases with input from the university librarian to specifically target the organization of services. This was an iterative process evolving over several months to result in the final search strategy. Articles already reviewed in the previous scoping review (4) were removed from the search as to not be screened again. However, the 10 articles that were included in the previous scoping review which addressed services for this population were screened for eligibility, and those fitting the new inclusion criteria were included in this current literature review for additional analysis.

**Table 1:** Search strategy for medline including keywords.

**Physical disability** (Physical* disab* or Physical* handicap* or Physically challenged or Physically disabled or Cerebral Palsy or Spina Bifida or Myelomeningocele or Meningocele or Spinal muscular Atrophy or Duchenne Muscular Dystrophy or Congenital Deformit* or Juvenile Arthr*).ab,kw,ti. Advanced search exp cerebral palsy/or exp spinal Dysraphism/or exp Meningomyelocele/or exp meningocele/or exp muscular atrophy, spinal/or exp Muscular Dystrophy, Duchenne/or exp disabled children/
AND
**Mental Health Disorder** (Mental* ill* person* or Mental health* or Psychiatric diagnosis or Psychotic disorder* or Brief reactive psychosis or Schizoaffective disorder* or Schizophreniform disorder* or Psychosis or Schizophrenia or Schizophrenic disorder* or Personality disorder* or ADHD* or Bipolar disorder* or Bipolar depression or Manic disorder* or Manic state* or Mania or Bipolar affective psychosis or Anxiety or Depression or Substance* abuse* or Substance* related disorder* or Eating disorder* or ASD or Autism or DSM).ab,kw,ti. Advanced search exp mental disorders/
AND
**Young adults and adolescents** (Child* or Adolescen* or Young adult*or teen*).ab,kw,ti. Advanced search exp youth/or exp adolescent/or exp young adult/
AND
**Healthcare services** (healthcare service* or service delivery or service delivery model* or organization of service* or rehabilitation or rehab* or care or occupational therap* or physical therap* or physiotherap* or transitional care or transition or model* or framework* or guideline* or service integrat* or coordinat*).ab,kw,ti. Advanced search occupational therapy/or physical therapy/or health services/or adolescent health services/or community health services/or health services for persons with disabilities/or mental health services/or student health services/or Transition to Adult Care/or “Continuity of Patient Care"/or “Delivery of Health Care"/

### 2.3. Selecting studies

#### 2.3.1. Inclusion criteria

The inclusion criteria for the selection of articles are based on our previous review (4) and have been adapted, where applicable, to meet the objectives of this current study:

1) A mean age of participants between 15 and 24 years old, a study population including at least 50% youth between ages 15 and 24 with specific results about this group, or the term *adolescents, youth,* or *young adults* used to describe their population, if no age is mentioned. The age limits correspond with the United Nations definition of youth (22).
2) A sample diagnosed with childhood-onset disabilities (pertaining to at least 50% of the total sample). Childhood-onset physical disabilities include cerebral palsy, spina bifida, muscular dystrophies, juvenile idiopathic arthritis, or other chronic physical disabilities that mainly affect movement and mobility, or children with special healthcare needs.
3) A focus on psychological problems, mental illnesses or symptoms of emerging mental disorders. These include anxiety, depressive, personality, psychotic and neurodevelopmental disorders such as autism, and their associated symptoms, as per the Diagnostic and Statistical Manual of Mental Disorders, 5th edition (23). In addition, studies were included if they focused on social or behavioral difficulties (with the addition of ADHD as a search term) as these were prevalent in the previous scoping review conducted by our team and could be a precursor to a mental illness diagnosis (4). There was also an addition of the personality disorder* search term to the previous strategy as it was listed by the stakeholder clinicians as a co-occurrence that they regularly came across in their work setting (see Table 1 for the full list of terms searched).
4) A focus on the organization of services, including if they reported a healthcare service delivery principle, model, or framework. Interventions were included if they described details pertaining to the context (e.g., which professionals are involved in the delivery).
5) Published in French or English (to accommodate authors' language fluency), between January 2000 and June 2021. While Lal and colleagues’ scoping review (4) had a lower limit of the year 2007, there were major shifts in thinking about healthcare reorganization happening before then. The WHO produced a World Health report (24) emphasizing that mental health care had been neglected for too long and was crucial to address in order to support healthy populations worldwide. This problem was also evident on more local scales, including in the province of Quebec, Canada, coinciding with the review of provincial healthcare service delivery highlighting lack of access to, and coordination of, care (25). Shortly after, Quebec's Ministry of Health and Social Services mental health reform mandated a reorganization of services and improvement of primary mental health care (26). Therefore, the lower limit was determined to be the year 2000. The WHO has since highlighted the importance of integrating mental health into general health facilities, encouraging a shift away from historical psychiatric hospital-based approaches to treatment (27).

#### 2.3.2. Exclusion criteria

The following exclusion criteria were applied: the article does not give sufficient and pertinent information to be analyzed; the document solely describes the prevalence of the conditions; the document describes services solely provided in the school setting (as they have distinct organizational structures and are funded and monitored by ministries outside of health and rehabilitation); the document does not describe the service program/model targeting both physical and mental health in enough detail. Reviews were not included, but their reference lists were hand-searched for relevant articles fitting the inclusion criteria. Non-peer reviewed articles (such as theses) were also excluded.

#### 2.3.3. Process of study selection

The searches were imported into Endnote ×7 and duplicates were removed. Articles were then uploaded to the Rayyan QCRI web application for the initial abstract screening to facilitate collaboration by enabling blinding of decisions to include or exclude between reviewers and labeling reasons for exclusion (28). First-level screening based on title and abstract was completed by two reviewers. They scanned 10% of the Medline articles independently and achieved 90% agreement on whether articles should be excluded, after which a discussion with a third team member helped to clarify the inclusion and exclusion criteria. The remaining articles were screened by one reviewer based on title and abstract, and reasons for exclusion after first-level screening were recorded. A subsequent full text (second level) screening of the retained articles was completed independently by two reviewers, and disagreements were resolved with discussion between the reviewers and additional members of the research team. Validation of all included articles and a random selection of 20% of excluded articles was completed by a third reviewer. Reasons for the exclusion of articles after full text screening were also recorded.

### 2.4. Charting the data

#### 2.4.1. Data charting process

**Figure 1:**
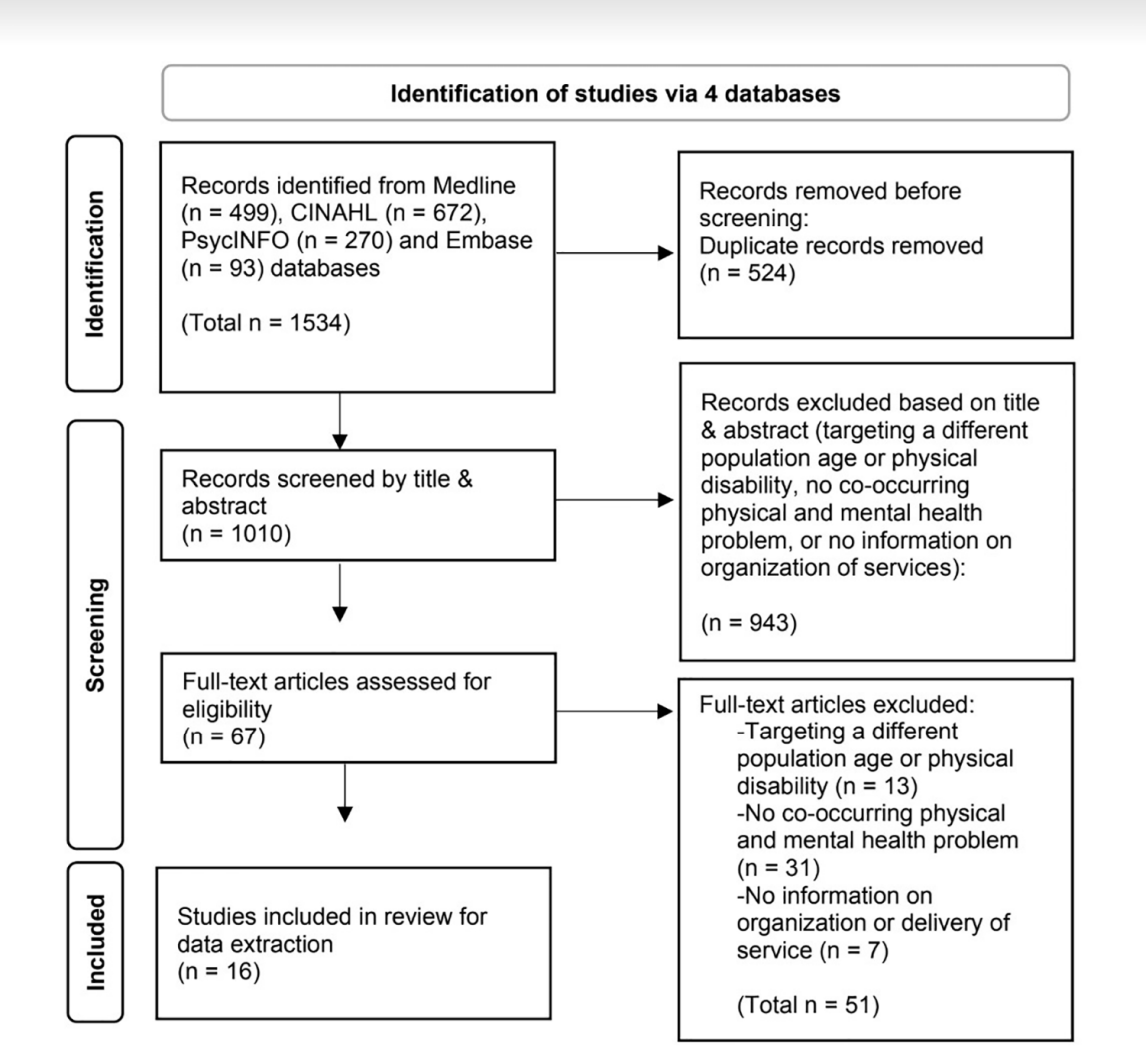
PRISMA flow diagram of included articles.

A flow diagram based on the PRISMA-ScR checklist guidelines (29) was completed to report the total number of sources of evidence screened, total number assessed for eligibility at each screening stage, reasons for exclusion, and total number of studies included in the final review (30, 31) as illustrated in Figure 1. A summary data extraction chart of the selected articles was created by the research team using Excel software and inspired by templates from previous scoping reviews (4, 21). Two reviewers independently extracted data, and the information was later merged and discussed together, and then again with a third reviewer. Only data pertinent to the main objectives of the scoping review was extracted (e.g., methods and results describing mental health or access or use of mental health services for youth with co-occurring disabilities).

#### 2.4.2. Data items

Data selected for extraction included the study characteristics such as the author(s), year of publication, title, country, study population, purpose of study or objective(s), research design, and main findings pertaining to the three research sub-questions (including key principles, services, models, and frameworks; see Table 2).

**Table 2:** Data extraction form with included articles.

Authors, Title, Year, Country	Study Population and Research Design	Purpose	Main Findings	Service Organization, Delivery & Needs	Key Components of Care and Models
**Bachmann et al.** Integrating children's services in England: national evaluation of children's trusts 2009 United Kingdom	147 managers and professionals working in the children's trusts for disadvantaged children with complex needs (lead children's trust managers and directors of child services) Prospective observational mixed methods	Describe and compare implementing programs integrating children's services in all 35 children's trust pathfinders (for 20% of children in England)	35 local areas were chosen as children's trust pathfinders to pilot coordinating and providing children's services. The national evaluation found that they had established a board for coordinating services, had a children and young people's plan, coordinated local and national budgets, wider scope of services, better coordination between agencies and outreach to communities leading to increased access to services (improved outcomes for families)	Multi-agency collaboration, pooled finances, information-sharing systems, shared geographical boundaries	Multi-agency, interprofessional work, shared budgets, shared information, streamlined referrals, training in complex care, key workers to coordinate, enthusiastic local leaders
**Berens et al.** Transition to Adult care 2020 United States	Children and transition-aged youth with CP Descriptive, conceptual	Examine topics and provide relevant resources about the transition to adult-based health care for individuals with CP		Structured approach including mental health screening, discussion with youth about psychosocial considerations (and who should be involved in those discussions)	Start planning the transition early, partner with family, shared decision making, transition supports in an electronic medical record, individual plan, transition primary care first, care coordinator, and systematic approach to evaluating psychosocial components
**Butler et al.** Shared decision making (SDM) among parents of children with mental health conditions compared to children with chronic physical conditions 2015 United States	Parents of children 2–17: (1) with a chronic physical illness but no mental health condition; (2) with a common mental health condition but no chronic physical condition; and (3) with comorbid mental and chronic physical conditions. Retrospective cohort cross-sectional study	Examine how parent reported SDM varies by child health status (physical illness, mental health condition, and comorbid mental and physical conditions) and examine the impact of medical home care on these differences	Mental health stigma, negative parental perceptions about mental health treatment options and complexity inhibit parent participation in SDM in child mental health care, but medical home care can help.	Implementing components of the medical home care	Shared decision-making (including behavioural interventions), partnerships between parents and providers, decision aids, workshops for parents, skills training for clinicians
**Colver et al.** How well do services for young people with long term conditions deliver features proposed to improve transition? 2018 United Kingdom	374 young people aged 14 years to 18 years 11 months who used child healthcare services in a range of United Kingdom locations, including 150 with type 1 diabetes, 118 with ASD and 106 with CP Longitudinal cohort + subsample interviews mixed methods	To describe whether service providers offer 9 beneficial features of transition services (from providers and youth with long-term conditions transitioning to adult services)	The 9 services (age-banded clinic, meet adult team before transfer, promotion of health self-efficacy, written transition plan, appropriate parent involvement, key worker, coordinated team, holistic life-skills training, transition manager for clinical team) were not well provided (even when services said they did, youth reported discrepancy). Many were against the written transition plans (more paperwork)	Many services did not provide the proposed beneficial features. The only features that were experienced by youth with CP was appropriate parent involvement and promotion of health self-efficacy	Age-banded clinic; meet adult team before transfer; promotion of health self-efficacy; written transition plan; appropriate parent involvement; key worker; coordinated team; holistic life-skills training; transition manager for clinical team
**Glassgow et al.** Behavioral Health Disparities Among Children and Youth with Special Health Care Needs 2017 United States	Children and youth with special healthcare needs including chronic complex conditions Conceptual perspective	To describe mental, emotional, and behavioral problems and disorders and disparities in mental health services among children and youth with special healthcare needs		Objective mental health assessments not common	Early mental health screening and intervention, family-centered approach
**Hanson et al.** *Experiences of employment among young people with juvenile idiopathic arthritis: a qualitative study 2018 United Kingdom	13 interviews and 3 focus groups (*n* = 9,4,3) with young people (16–25 y) and adults (26–31 y) mean age 22 with JIA and interviews with (*n* = 9) health professionals Qualitative description	To explore expectations and experiences of employment among young people with JIA and the role of health professionals in promoting positive employment outcomes	Lower employment rate for those with JIA due to physical and psychological impacts of the disease like pain, which was not evident for employers, and they had anxiety with regards to disclosure (or not) regarding their disability and attitudes of their employer	Multidisciplinary team involvement in promoting employment, psychosocial interventions, and few mental health professionals available. Training for professionals on complex care is needed	Disease management, flexible convenient care, information, emotional and social support, skills training, advocacy
**Houtrow et al.** Profiling health and health-related services for children with special health care needs with and without disabilities 2011 United States	CSHCN with disabilities (one age group 14-17 years) Secondary review of national survey data	To compare health services characteristics for children with special health care needs with and without disabilities and examine factors associated with unmet need	CSHCN with disabilities had higher rates of need and unmet need than other CSHCN for specialty care, therapy services, mental health services, home health, assistive devices, medical supplies, and durable medical equipment, despite more severe health conditions	More unmet needs (including accessing mental health services) for those with disabilities, little coordination of care	Early mental health screening and intervention, medical home model
**Lindsay et al.** *Enablers and barriers of men with Duchenne muscular dystrophy transitioning from an adult clinic within a pediatric hospital 2017 Canada	16 participants (7 clinicians, 5 parents, 4 youth) with Duchenne muscular dystrophy Qualitative	To explore the enablers and barriers of clinicians, young men, and parents as they transition from an adult DMD clinic within a pediatric hospital to an adult health facility	Clinicians, youth and their parents experienced several enablers and barriers in transitioning to an adult health care center. Clinicians reported that structural factors (leadership and advocacy) supported the transition. Clinicians and parents found that the availability and continuity of care both enabled and hindered the transition. Parents and youth found adjusting to the model of adult care and accessing resources challenging. All reported difficulties maintaining mental health for youth with DMD transitioning to adult health care	Leadership, advocacy, inter-agency partnership, no cross-appointed clinician, holistic comprehensive model of care only in pediatrics, support for relationships, sexuality and depression was missing	Emotional support (peer and clinical), family involvement, inter-agency collaboration, cross-appointed clinician, funding, leadership advocacy, staff training
**Park et al.** Health care services and the transition to young adulthood: challenges and opportunities 2011 United States	Adolescents with special healthcare needs in transition to adult care Conceptual review paper and national survey analysis of key outcomes	To describe a role for integrated health care services in transition		There are gaps in access and quality of care for those with mental and physical health problems. There is inadequate coverage of needed adolescent mental health services, mental health services are put apart in separate systems, a shortage of mental health professionals trained to serve adolescents	Medical home model, interprofessional work, early screening, shared decision-making, training in complex care, self-management of chronic conditions (increasing responsibility), adequate insurance, community-based, considers transitions
**Roman et al.** Analysis of Care Coordination Needs for Families of Children with Special Health Care Needs 2020 United States	Children and youth (age 0-21) CSHCN served by the Medical Home Initiative program at the Center (2,682 participants) Analysis of service use data	To identify the diverse services needed/requested by families of CSHCN and identify the specific CC efforts for different diagnoses	The most frequently required sectors across the study population were education, financial, medical/dental, social connections, and advocacy. Children diagnosed with autism spectrum disorder had the highest needs across all sectors. Most CSHCN and their families use a substantial amount of CC time and effort to secure services from diverse sectors	Coordination of care that is patient- and family-centered, assessment-driven, team-based, with collaborations across multiple sectors, but many associated challenges (ex. lack of a universal release of information, incompatible electronic health record systems, and regulatory requirements)	Assessment, family-centered planning, implementation, evaluation, monitoring, support, education, and advocacy. Social workers as coordinators. Multi-agency cross-sector collaboration (health and education).
**Scratch et al.** Mental Health Care in Pediatric Rehabilitation Hospitals: A Biopsychosocial, Collaborative, and Agency-based Service Integration Approach 2020 Canada	Models and guidelines (Canadian and international) relating to mental health services in pediatric rehabilitation hospitals Conceptual environmental scan of best practice guidelines	To describe a practical and holistic framework for pediatric rehabilitation hospitals to meet the health care needs of children and their families		Two-phase approach: build staff capacity for MH (referrals to internal MH team) to eventually have MH staff fully integrated into rehabilitation team	Interdisciplinary healthcare team, staff capacity, screening & identification of biopsychosocial factors, efficient referrals in-house to MH specialists, holistic care plan. Assessment of family function and consider assessing family mental health **Biopsychosocial, Collaborative, and Agency-based Service Integration Approach** inspired by biopsychosocial model of care, collaborative mental health model and psychiatric consultation service models
**Tong et al.** *Consumer Perspectives on Pediatric Rheumatology Care and Service Delivery 2013 Australia	37 parents and 13 adolescents aged 14-20 with JIA Qualitative	To elicit parental and adolescent perspectives on pediatric rheumatology care and service delivery and to describe the impact of this process on a proposed model of care addressing pediatric rheumatology service delivery	Five main themes were identified for the model and was extended to include consumer-focused concerns. A well-coordinated network of services, timely and accurate information about the illness, treatment and support services, adequate pharmacy support, and school-based advocacy are proposed to be needed to ensure pediatric rheumatology services that are accessible and responsive to the needs of patients and their families	Optimized service efficiency, transitional care, psychosocial support, informational needs, school-based support, and advocacy	Integrated clinics, faster diagnosis, peer and sibling support, psychological services, family functioning, financial aid, access to information, school-based support, and advocacy
**Van Dongen et al.** A protocol for interagency collaboration and family participation: Practitioners’ perspectives on the Client Network Consultation 2018 Belgium	23 clinicians working with children having complex healthcare needs in child disability or mental health care settings Exploratory	To develop and evaluate a standard protocol on the structure, content, and impact on interagency collaboration with family involvement based on the wraparound principles (collaborative planning process for individualized treatment plan)	Focus groups evaluated the CNC by eliciting practitioners’ views on the structure, content, and impact of collaborative interagency protocols with family involvement. Thematic analysis revealed four core themes: (1) Empowering the child and the family; (2) Utilizing the strength of the collective; (3) Being considerate versus constructive a dilemma for participants in CNC; and (4) The structure of a protocol offers opportunities and challenges	Client Network Consultation 3 phases: preparation (conversation with child and parents to identify strengths, needs, cultural elements, and long-term goals), individualized care plan development (care manager for coordination) and implementation of plan and care team (progress reviewed and changes can be made to plan). The structure also includes strategies to make conversations between families and practitioners possible.	Inter-agency collaboration, care manager for coordination, wraparound principles (collaborative planning process with family for individualized treatment plan and discussions, strengths-based, adapts over time, culturally competent) **Client Network Consultation** – Phase 1 - Engagement & Team Preparation – Phase 2 - Care Plan Development – Phase 3 - Implementation of Care Plan
**Warfield et al.** Unmet need and problems accessing specialty medical and related services among children with special health care needs 2006 United States	2,220 families of children under 18 (mean age 8.9) with special healthcare needs 20-state survey	To extend what is known about parent reports of their child's need for specialty medical services, unmet need, and specific types of access problems among children with special health care needs	Unmet needs were greater for older children and those with complex problems, and access to mental health and home services were less likely	High need and limited access to mental health services, need more outreach and screening	Care coordination, medical home, family-centered care
**Witt et al.** Mental health services use among school-aged children with disabilities: the role of sociodemographic, functional limitations, family burdens, and care coordination 2003 United States	4,939 community-dwelling children with disabilities, ages 6 to 17 years Analysis of National Health Interview Survey Disability Supplements (NHIS-D)	To examine the use of mental health services and correlates of receiving services among community-dwelling children with disabilities	Only 41.8% of those with disabilities and poor psychosocial adjustment accessed mental health services	Low access to mental health services, but providers involved in care coordination improved this. Engaging family and healthcare professionals in the process	Care coordination, multi-agency collaborations (family-health-education), family-oriented care, family and staff training, reimbursement for coordination of care services, having a coordinator
**Woodward et al.** *Assessing the health, functional characteristics, and health needs of youth attending a noncategorical transition support program 2012 United States	198 parents of youth with special health care needs (including CP 36%, SB 10%, mean age 17.5) attending the transition clinic for chronic conditions Local survey and comparison to national survey of CSHCN	To assess the health, functional characteristics, and health care service needs of youth and young adults with special health care needs attending a comprehensive, noncategorical transition program	Youth attending our transition program had more functional limitations, poorer reported health status, different diagnosis distribution, and higher levels of needed health services. Few parents identified needs for other recommended adolescent preventive services. Noncategorical transition programs currently in development will need the staff and skills to address the multiple needs of a medically complex population of patients	There were unmet needs for durable medical equipment, therapies, and medical supplies. Overall a structured approach, service delivery model and skilled staff are required	Nurse care coordinators, social work care coordinators, multidisciplinary team, staff training for complex care

* Articles from earlier scoping review conducted by our team (4).

#### 2.4.3. Critical appraisal of individual sources of evidence

Scoping reviews do not typically include a critical appraisal of the evidence as they describe rather than analyze and report (18). Additionally, given the large variability of study designs and research approaches likely to be found in a scoping review, a critical appraisal is challenging, and it is unclear whether it has an impact on the uptake of results (16), therefore it was not undertaken in this review.

#### 2.4.4. Consultation

Consultation with stakeholders brings valuable input with regards to the needs of youth, families, and clinicians as well as the clinical realities faced and their wishes for integrated health and rehabilitation services (24). A first meeting was held in consultation with three clinician stakeholders (from physical rehabilitation teams) to contribute ideas pertaining to additional literature to investigate and review the search strategies. In a second interaction, stakeholder consultants (one clinician and one youth) reviewed preliminary findings to ensure that the results were coherent and presented in a meaningful way, and the suggestions generated were integrated.

### 2.5. Collating, summarizing, and reporting the results

Counts, proportions, and tables were used to synthesize study characteristics such as country, study design, and study population characteristics (e.g., type of diagnosis, age). Findings related to mental health services among the study population were coded into themes of principles through inductive content analysis (32). Main ideas were extracted based on the 3 sub-questions (How are these services organized and delivered?; What are the key principles of existing models of service delivery?; What is known about the effectiveness of identified models and principles?), and keywords were determined based on if they were strategies or structural elements that facilitated service organization. These keywords were transposed into a table and organized into principles across articles by two team members independently, then compiled by grouping similar terms together. The key principles were described (with a comprehensive definition and examples from the articles for each) and were grouped into overarching categories of themes. The frequency of principles addressed across articles was tallied and presented in percentages. The table was then reviewed by two senior team members independently for validation, and consensus (on the key principles, themes, definitions, and their frequency) was reached through discussions with the whole team.

## 3. Results

### 3.1. Overview of studies reviewed

A total of 1,534 articles were retrieved from the 4 databases, and 1,010 remained after removing duplicates. After first-level screening, 943 articles were excluded because they did not meet the established inclusion and exclusion criteria (as they were either targeting a different population age or physical disability, had no co-occurring physical and mental health problem, or included no information on organization or delivery of services). Next, 67 articles underwent second-level screening, and 16 documents were selected for data extraction (33–48). The 51 articles that were excluded during the second-level screening were either targeting a different population (in terms of age or physical disability), did not explicitly address a co-occurring mental health problem, or did not include enough information related to the organization or delivery of rehabilitation or healthcare services. A PRISMA flow diagram is presented in Figure 1 (31).

Details pertaining to the 16 articles that were retained for data extraction are provided in Table 2. Articles originated from five different countries, with the largest proportion (9/16) published in the United States, followed by 3 in the United Kingdom, 2 in Canada, 1 in Belgium and 1 in Australia. The 16 selected articles used a variety of designs, including 6 quantitative cross-sectional studies, 4 qualitative studies, 4 conceptual papers and 2 mixed methods studies. The largest portion of the articles focused on a general group of youth with complex or special healthcare needs or a mix of childhood-onset physical conditions (including cerebral palsy, spina bifida and muscular dystrophy; *n* = 11), followed by study populations consisting entirely of youth with cerebral palsy (*n* = 2), juvenile idiopathic arthritis (*n* = 2) or muscular dystrophy (*n* = 1).

### 3.2. Service organization and delivery approaches

When looking at how services were organized and delivered (research sub-question 1), several (*n* = 6) articles (33–35, 43–45) described what was currently being done in practice to address the high need for mental health services. These will be detailed further below. In contrast, most (*n* = 10) articles highlighted the lack of structure, organization, and access, and focused on recommending future directions to address pressing needs. Some reported that objective mental health assessments were not commonly used with youth with chronic conditions or special healthcare needs (36, 37), and that more outreach and screening was needed in populations who already have diagnosed physical disabilities (46, 47). Others highlighted missing structural elements such as lack of a universal release of information on charts, incompatible electronic health record systems across sites, regulatory requirements (42) and structured approaches (48) that brought challenges when attempting to share information between collaborating centers wanting to work efficiently. Gaps in access and quality of mental health care for those with co-occurring mental and physical health problems were prevalent, and the various healthcare services needed were seldom coordinated for populations with complex healthcare needs such as seeing a psychologist or receiving therapy (39, 41). Comprehensive organization of care was even less evident after the transition from pediatric to adult settings, and support for relationships, sexuality and depression was missing altogether (40). Overall, an inadequate coverage of needed adolescent mental health services was expressed, either being made available in completely separate systems which were hard to access (i.e., not where the youth was already receiving care), or not at all available (41).

### 3.3. Formal models of service organization and delivery

Only two formal models of service delivery for co-occurring physical and mental health problems were described across the retained articles (43, 45). The first model found through this review was the Biopsychosocial, Collaborative, and Agency-based Service Integration Approach, developed in Canada and presented in a conceptual paper by Scratch and colleagues in 2020 (43). It described a 2-phase process to include and fully integrate psychiatry services within a pediatric rehabilitation hospital treating developmental and other chronic disabilities. Recommendations from clinical guidelines and national strategy documents were combined to develop a service integration approach built into a multidisciplinary rehabilitation setting with clinicians from varied backgrounds (ex. psychology, physical therapy, occupational therapy, speech language pathology, social work, nursing). The first phase of the model targets building staff capacity around recognizing and treating mental health needs in children and their families including structured cross-referral from the rehabilitation team to a newly formed internal mental health team who can provide integrated treatment recommendations and support the rehabilitation team in providing comprehensive care. Phase II focuses on creating and evaluating a comprehensive model of health care provision where the capacity of staff on the rehabilitation team in relation to mental health and psychosocial needs increases, and mental health specialists are fully integrated as interdisciplinary rehabilitation team members. The second model of service delivery found through this review was the Client Network Consultation (CNC) model developed in Belgium by Van Dongen and colleagues in 2018 (45). Described in a qualitative paper, specialist clinicians from different teams (including child welfare, psychiatry, and rehabilitation) participated in 3 focus groups to inform and evaluate an interagency collaboration in mental health care for children with complex healthcare needs. It includes three phases (engagement and team preparation, care plan development, and implementation) based on the wraparound principles (including collaboration and cultural competency) and details a collaborative planning process with staff, families, and their support network to create an individualized strengths-based treatment plan which can adapt to needs over time. They came up with four core themes (Empowering the child and the family; Utilizing the strength of the collective; Being considerate vs. constructive, a dilemma for participants in CNC; The structure of a protocol offers opportunities and challenges) which illustrate clinicians' perspectives towards putting the model into practice.

### 3.4. Key principles for service organization and delivery

**Figure 2:**
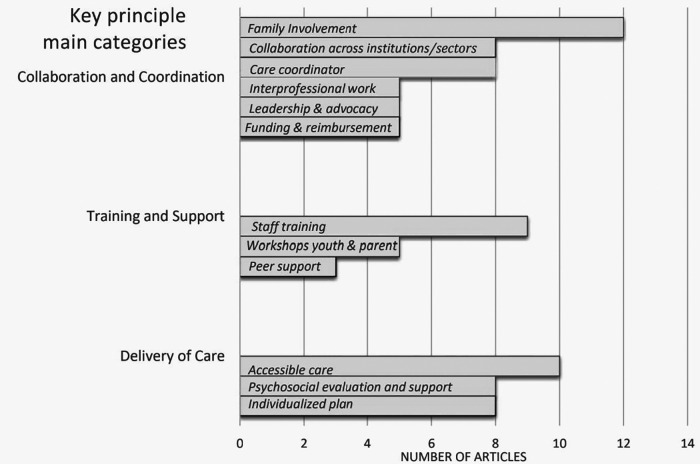
Key principles across articles.

Key principles within these two existing models of service delivery as well as the other included articles were extracted to address research sub-question 2. Across the 16 articles, there were 12 key principles that were synthesized *via* qualitative content analysis and grouped into 3 main categories or themes, namely: Collaboration and Coordination; Training and Support; and Delivery of Care (see Figure 2). Each article encompassed between 2 and 8 key principles spanning the different categories (see Table 3). As for the two models described above, the Biopsychosocial, Collaborative, and Agency-based Service Integration Approach included 6 and the CNC model included 5 key principles.

**Table 3:** Key principle themes and subthemes with definitions across articles.

Theme	Key Principle	Definition	Articles which included the key principle
Collaboration and coordination	Family involvement	Involving the family and youth in shared decisions about care, consulting with all stakeholders	(34–37, 40–47) *n* = 12
	Collaboration across institutions/sectors	Collaborating across several institutions spanning different sectors (i.e., education, health, and social services) and settings (home, school, community)	(33, 34, 38, 40, 42, 44, 45, 47) *n* = 8
	Care coordinator	Also referred to as a key worker, a designated person who coordinates with family, youth and treating team, familiar with the case and with relevant resources	(33, 34, 36, 40, 42, 45, 47, 48) *n* = 8
	Interprofessional work	Team including different types of healthcare professionals working together	(33, 36, 41, 43, 48) *n* = 5
	Leadership and advocacy	Having someone or a group of people advocate for changes to improve services	(33, 36, 38, 40, 44) *n* = 5
	Funding allocation and service reimbursement	How money is allocated to fund different programs and services, whether it is covered by insurance	(33, 40, 41, 44, 47) *n* = 5
Training and Support	Staff training	Workshops for clinicians so they feel well prepared to work with clients having mental health problems	(33, 35, 38, 40–43, 47, 48) *n* = 9
	Workshops for youth and parents	Information delivered in group settings to help understand the complexities of care and be better prepared to make decisions	(35, 36, 38, 41, 47) *n* = 5
	Peer support	Access to support and guidance from others with lived experience	(38, 40, 44) *n* = 3
Delivery of Care	Accessible care	Having flexible and convenient care provided in a customized way to adapt to client needs, working towards integration	(33, 34, 36, 38, 39, 41, 43–46) *n* = 10
	Psychosocial evaluation and support	Formal evaluations to assess mental health, diagnose	(34, 37, 39–44) *n* = 8
	Individualized plan	Having a formal plan established and written out with team, considering culture and personal values/beliefs	(33–36, 41, 43, 45, 46) *n* = 8

Collaboration and Coordination: There were 6 key principles that fit into this first main category, namely *Family involvement (present in 75% of articles); Collaboration across institutions/sectors (50%); Care coordinator (50%); Leadership and advocacy (31%); Funding allocation and service reimbursement (31%);* and *Interprofessional work (31%)*. The key principle of *family involvement* meant involving the family and youth in decisions about their care, thus ensuring consultation with all key stakeholders. Twelve articles discussed the importance of regular family involvement and integration into the team, making it the most common principle across articles (34–37, 40–47). Included in this are the concepts of shared decision-making, where families are true partners in the process (34, 35, 41) and family-oriented care (36, 42, 46, 47). One example of shared decision-making, which included discussing treatment options, providing information and opportunities to ask questions, and exploring parents' ideas of management, was presented in a retrospective cohort cross-sectional survey by Butler and colleagues (35), but they found that it was much more complex to put into practice for those with mental and physical comorbidity vs. just one or the other. In a quantitative study by Roman and colleagues (42), pediatric care coordination was described as a patient- and family-centered activity designed to meet the needs of youth while improving the caregiving capabilities of families as a whole, making their involvement crucial.

*Collaboration across institutions* related to (informal or formal) partnerships between several institutions, sometimes even spanning beyond healthcare into different sectors (i.e., education and social services) and settings (home, school, community). Eight articles pertained to this key principle and highlighted how coordination was crucial (33, 34, 36, 40, 42, 45, 47, 48). For example, this was done by building collaborations between the family, medical, psychological, and educational systems of care to improve access to services (47), or by creating inter-agency partnerships between different pediatric and adult healthcare settings to facilitate a smoother transition during this particularly precarious time for youths' mental health (40, 45). Actions such as sharing clinical information across healthcare sites (33, 38, 42, 44) and using a transition registry when moving to adult care (34) supported these collaborations.

The key principle of *Care coordinator*, also sometimes referred to as a key worker (33, 36) or cross-appointed clinician (40), was described as a designated person who serves as an easily accessible point of reference, coordinating with the family, youth and treating team, and being familiar with the case and with relevant resources. Eight articles mentioned the use of a care coordinator in their settings (33, 34, 36, 40, 42, 45, 47, 48), with two specifying that nurses (48) and social workers (42, 48) were particularly well-suited for this position. Many articles specified that the role was filled by clinical providers (34, 40, 42, 47, 48), while others held a management position (45). Colver and colleagues (36), in a mixed methods study following 374 young adults with disabilities longitudinally from different services as they transitioned to adult care, discussed the role of a key worker as one of nine proposed beneficial features. However, only half of the young adults confirmed experiencing this feature when the service stated that it was provided (which was only in 43% of services across conditions, and 15% for those receiving services for cerebral palsy specifically).

The principle of *Leadership and advocacy* pertains to a person or a group of people who advocate for changes to improve services. Five articles described leadership qualities or actions that contributed to improved care, including the importance of advocating when barriers to accessing care were present (33, 36, 38, 40, 44). In a large mixed methods study spanning services across the United Kingdom, Bachmann and colleagues (33) highlighted the necessity of enthusiastic local leaders to bring about change, in addition to local cultures and experiences of cooperation that could overcome organizational and professional barriers.

The principle of *Funding allocation and service reimbursement* was addressed in five articles (33, 40, 41, 44, 47). It included how money is allocated to fund different programs (40) and professionals, such as ensuring adequate reimbursement for providers who actively coordinate the care of children with disabilities as found in an American national survey of 4,939 children and youth with disabilities (47). It also examined whether services are covered by insurance (41, 44) which is of particular importance in the United States. Shared budgets were a strategy that allowed for better coordinated care across different sites within the healthcare system and reduced barriers to inter-institutional collaboration as identified by 147 managers and policymakers in the United Kingdom (33).

Finally, *Interprofessional work* involved being followed by a healthcare team made up of different types of professionals working together. Five articles explicitly stated the importance of interprofessional work (33, 36, 41, 43, 48), and encouraged increased capacity in mental health screening and identification of biopsychosocial factors as well as the possibility for efficient referrals to mental health specialists in-house. Three teams conducted large studies in the United Kingdom (33, 48) and the United States (36) and discussed the integration of specific elements of interprofessional work, highlighting that many were not well integrated into practice. Only about half (56%) of young adults (*n* = 304) in the American study reported being adequately followed by a coordinated team of professionals (36).

Training and Support: There were 3 key principles that related to the category of training and support resources for youth, families, and the care team, including *Staff training (56%); Workshops for youth and parents (31%);* and *Peer Support (19%).* Nine articles brought up *Staff training*, which in the context of this review specifically referred to workshops for clinicians to enhance their skills to work with clients having mental health problems, in contrast to strictly physical rehabilitation (43). These workshops should be provided in the workplace to facilitate team training (33, 34, 40, 42, 47, 48). Park and colleagues (41) described the importance of having training in areas such as primary care and new models of interdisciplinary mental and behavioral health to help address the shortage of clinicians trained to serve adolescents. Hanson and colleagues (38) echoed this, stating that there were unmet training needs for professionals in the United Kingdom working with a population having juvenile idiopathic arthritis (JIA) regarding childhood-onset chronic conditions and their impacts on their mental health and emphasizing that training on complex care was needed.

*Workshops for youth and parents* encompassed information provided in various forms (such as in-person group sessions) to better inform service users and their families about the complexities of care so they could be better prepared to make decisions. Five articles suggested that workshops for youth, such as holistic life skills training (36), and parents (35, 47) were important. Disease self-management for chronic conditions was seen as useful and empowering, for example in a group of youth living with JIA (38). A conceptual paper by Park and colleagues (41) highlighted that adolescents with special healthcare needs should assume increasing responsibility to manage their health with appropriate clinical guidance, and that providing confidential care also enabled development of skills in self-disclosure, especially for sensitive topics that frequently emerge during adolescence, such as sexuality and substance use.

Finally, *Peer support* was described as having access to support and guidance from others with similar lived experience. Three articles, based on qualitative investigations, encouraged peer support groups for youth with disabilities to help them navigate the associated complexities (38, 40, 44). To illustrate, in interviews with 16 clinicians, parents and youth with Duchenne Muscular Dystrophy in Canada, all participants reported the difficulties of maintaining mental health for these youth transitioning to adult health care and encouraged peer support groups to help deal with isolation and depression (40). Similar results were found regarding the need for emotional and social support from peers when interviewing 37 parents and 13 adolescents with JIA in Australia (44), and through interviews and focus groups with 38 clinicians and young adults with JIA in the United Kingdom (38).

Delivery of Care: Finally, there were 3 key principles pertaining to the elements of care delivery, including *Accessible care (63%); Psychosocial evaluation and support (50%);* and *Individualized plan (50%)*. *Accessible care* is related to having flexible and convenient care provided which could be adapted and customized to the client's needs, working towards a seamless integration of services. Ten articles described how accessible care could be provided (33, 34, 36, 38, 39, 41, 43–46), with 3 suggesting a medical home which brings the services to the family directly and facilitates partnerships with parents (39, 41, 46). Based on qualitative findings from focus groups conducted by Van Dongen (45), care must be strengths-based, able to adapt over time, and culturally competent. Flexible healthcare scheduling was suggested by youth with JIA receiving services in specialized programs throughout United Kingdom hospitals to ensure convenience for the client so they could attend their various appointments while still maintaining their regular routines such as going to school (38).

*Psychosocial evaluation and support* pertained to formal evaluations to assess mental health and screen for, and diagnose, psychiatric disorders early. Eight articles reported on the importance of specialized psychosocial care, and many emphasized early screening to address concerns before they worsen (34, 37, 39–44). Tong and colleagues (44), *via* interviews and focus groups, brought to light consumer (adolescent and parent) views on how combined clinics for integrated care could lead to a faster diagnosis of mental illness in a population followed in pediatric rheumatology clinics in Australia. Glassgow and colleagues (37), in their conceptual paper, stated that early identification in children and youth with special healthcare needs across the United States by way of integrated screening into primary care followed by mental health intervention is key.

Lastly, the *Individualized plan* was described as having a formal plan written out in collaboration with the clinical team, user and family which considers the client's culture and personal values/belief systems. Eight articles described how they included formal individualized plans in their care delivery built around the needs of the child and their family (33–36, 41, 43, 45, 46). The use of decision aids (in-house guidelines used to help plan treatment) was suggested by Butler and colleagues (35) in a national survey of over 21,000 parents of children with physical and/or mental health problems across the United States. Having a formal plan in place to organize care was particularly important when anticipating transitions from the pediatric to adult care settings (36, 41). Considering local cultures (33) and providing culturally effective care (46) were also emphasized when planning care delivery for children having complex healthcare needs.

### 3.5. What is known about the effectiveness of identified models or principles

Very little information was found about the effectiveness of identified models and principles (research sub-question 3), with only three articles mentioning that efficacy was not yet assessed. The CNC model developed by Van Dongen and colleagues (45) is the only study in this review that evaluated (by qualitatively examining) the experiences and perspectives of clinicians on implementing a model (or key principles) using focus group methods. This was done with specialist clinicians who had implemented it in their clinics, eliciting their views on the structure, content, and impact of collaborative interagency protocols with family involvement. They mentioned several strategies (including making tasks and expectations clear, creating a positive and structured environment, working collaboratively with specialist colleagues) as well as challenges (such as navigating time constraints to put the protocol in place, being transparent with the child and family when discussing delicate matters, and accepting their professional limitations).

## 4. Discussion

This review generated key principles for organizing health and rehabilitation services for youth with a range of physical disabilities who also presented with mental health or behavioral challenges.

### 4.1. Principal findings

Only two models of service delivery were described across the retained articles, this being despite the acknowledgement that formal models are important guides for organizing complex care delivery (48). Several of the principles generated in this synthesis were present within the two formal models of service delivery. Both included the principles of *Family involvement, Individualized plan,* and *Accessible care.* The model proposed by Scratch and colleagues (43) additionally included *Interprofessional work, Staff training,* and *Psychosocial evaluation and support*, whereas the CNC model (45) also included *Collaboration across institutions/sectors* and *Care coordinator*. The 12 key principles described in this paper can be used in practice in varying combinations and can be drawn upon to tailor to specific clinical contexts. They can also guide the development of models of service organization and delivery that are relevant for various settings. Some of the principles described are well known and well documented in the literature, such as *Care coordinator* (49), while others are more unique and worth reflecting upon further, as detailed below.

*Staff training* was prevalent as a principle, highlighting that many clinicians felt unequipped and unprepared to address mental health problems when working with youth having physical disabilities. Needed healthcare services such as receiving therapy were seldom coordinated for populations with complex healthcare needs (39, 41). This can be partially explained by a finding from Hanson and colleagues' article (38) describing the low availability of professionals with mental health training working in physical rehabilitation settings with youth, for example those with JIA. The ability to identify mental health problems is crucial to early intervention, though little information is available in the physical rehabilitation and health services literature to guide mental health training options for their multidisciplinary teams. Whitehurst and colleagues (50) identified this gap in a similar population and created an in-house, 2-day training program (including information on mental health diagnoses, assessments, interventions, etc.) offered to all frontline staff in a residential school in the United Kingdom catering to young people between the ages of 6 and 19 with severe intellectual disabilities. Staff must have the necessary knowledge and screening or assessment tools and feel competent in identifying and addressing emerging mental health problems (or referring to other professionals) so that the youth and their families have direct access to required services (51).

*Accessible mental health services* are an essential component to comprehensive care, especially considering growing needs in youth mental health (52). The findings from this synthesis complement what was found in a previous scoping review (4), namely the 4 themes regarding access to services. Indeed, studies from that review found that few participants having mental health problems were receiving care for it, and there was a lack of or delay in help seeking and receiving care influenced by stigma, which in turn contributed to feelings of anxiety and impacted decisions to disclose their disability to others (4). In addition to these noted challenges, flexibility of services that could be delivered at a convenient time and location for the youth and their family (either integrated into the services they were already receiving or provided in the community or at home) was recommended. Adaptability over time to consider changing contexts and realities was also suggested.

The importance of family involvement is well-known in rehabilitation literature (53). Twelve of the retained articles emphasized the importance of family involvement, making it the most common key principle found in this review. Beyond being present for the care of their child, a recent scoping review confirms that families of children with neurodisabilities play a key role in delivering interventions to address comorbid mental health or behavioral problems (54), though training programs may vary in their aims, degree of involvement, content, and delivery methods. The associated burdens on family members should therefore be considered. The family and caregivers also need mental health support and services, delivered as a component of services for young people with disabilities (55). In addition to peer support for youth with physical disabilities, which is acknowledged by clinicians and youth as a potentially helpful resource and a way to reduce isolation (4, 55), peer support for their families is also very helpful. A recent study looking at families of children with neurodisabilities found that peer support (combining self-reflection and emotional expression) provided empathy and bridged communication between families and service providers when navigating complex healthcare systems which had a significant positive impact (56). Indeed, parents from included studies in this synthesis often felt overwhelmed and helpless, particularly when their child experienced severe depression, and emphasized the importance of accessing psychological intervention for themselves (44). Providing support to families can therefore ensure that they are capable of being there and forming a stronger support network for their child throughout the journey.

The literature included in this synthesis mainly discusses needs for better organization and delivery of health and rehabilitation services. Therefore, the challenges associated are quite clear, but the solutions are less obvious as there are many factors to consider. Every institution works in its own way, even within the same country, province, or city (57). Organizational culture emerged as important to consider in terms of cooperation, trust and values within, and across sites (33), and would be worth exploring further. Examining interagency collaborations and their dynamic interactions can help provide a better picture of the complete system of care. At the individual level, it is important to look at frequent users of rehabilitation services from a holistic perspective in order to address all of their needs over time (58). Having *individualized plans* that are not only holistic and created collaboratively with the user and family, but also accessible to relevant professionals and teams within and across organizations as necessary (such as *via* electronic records) could help with a better continuity of care. Different programs and institutions are encouraged to collaborate and communicate (such as with the help of *care coordinators*) to avoid gaps in care and complement each other with their strengths and specialties. It may also be useful to reduce the silos and have more flexibility within each program, not solely focusing on physical rehabilitation aspects of a disorder but instead having programs for youth with physical disabilities run by a *multidisciplinary team trained* to address multiple facets and challenges that come with living with a disability, which would include having mental health specialists integrated into the rehabilitation teams (43). The WHO is currently developing a Package of Rehabilitation Interventions which will focus on evidence-based strategies organized by age that will aid health ministries in planning, budgeting, and integrating rehabilitation interventions into health systems (59). Better integration of care will be critical to achieving universal health coverage and reaching as many individuals and families as possible to support health and wellbeing (12).

### 4.2. Identified gaps in current knowledge and future directions for research

As very few articles described formal models of organization and delivery of services, this is an area for continued research. Intensive services and treatment programs for this population are also uncommon given the complexity and diversity of the target population, and their effectiveness has not been previously reported. However, some literature is beginning to emerge on this topic. An American study recently described an intensive day treatment program for children with co-occurring chronic medical disease and emotional problems with 175 children followed over 3 years and reported significant improvements in depression and anxiety among others (60). A research protocol was also recently published in Canada on the impact of a transition readiness program for youth with physical health conditions in the presence of a mental health comorbidity, to explore the experiences of adolescents and young adults with co-occurring conditions as they exit pediatric services (61). More studies such as these with a focus on mental health are needed to advance rehabilitation research.

Effectiveness of current and future models and principles of service organization and delivery should also be assessed across different contexts. Although the principles discussed in this synthesis were not directly tested in their respective articles, they are based on previous research in the field. Now that co-occurrence of mental health problems and physical disabilities is acknowledged, the described principles can be evaluated with this complex population specifically.

### 4.3. Strengths and limitations

Strengths of this review include revealing a true gap in this specialized area *via* a comprehensive search strategy across multiple databases, systematic data extraction, clear eligibility criteria and focus on the organization of services for co-occurring physical disabilities and mental health problems. However, the heterogeneity in methodological, participant, outcomes and results resulting from the review's inclusive approach diminished the potential for meaningful comparisons across articles. The targeted population was youth with childhood-onset physical disabilities that were identified as having primarily motor problems, though broadening our scope to include other disabilities (e.g., neurodevelopmental) could have yielded additional results to better understand the landscape of complex rehabilitation service delivery for co-occurring problems. Furthermore, we included only scientific studies, while gray literature could have also provided rich data. Finally, most of the studies were carried out in high-income settings, while up to 80% of people living with disabilities reside in low- and middle-income countries (62) for which most evidence lacks representation and generalizability and which may not be feasible in resource-limited settings. This also implies that there may be sociocultural influences on attitudes regarding mental health and service trajectories that are yet to be explored.

## 5. Conclusion

This scoping review maps the current literature and synthesizes emerging principles to guide improved service delivery for this population as well as models of collaborative healthcare delivery described in the literature. A genuine disconnect between empirical evidence on prevalence of mental health problems among those with physical disabilities and the available services for youth with complex needs was observed, emphasizing an urgency for increased inclusion in clinical research. The findings from this review may lead to highlighting key attributes to include in a proposed model of service delivery (for replicability), with the potential to improve access and overall efficiency of services for youth across Canada and abroad, as well as inform the development of new programs to support these complex cases. This scoping review also identifies several gaps in the current literature (including formal integrated models of care and their effectiveness) that can inform future lines of inquiry in health and rehabilitation research.

## 6. Protocol registration and publication

The current protocol was registered online in Open Science Framework in December 2021 (19). Preliminary results were presented at national (Canadian Association of Occupational Therapists) and international (European Academy of Childhood Disability) scientific conferences in May 2022.

## Data Availability

The original contributions presented in the study are included in the article, further inquiries can be directed to the corresponding author.
